# Exercise and hyperuricemia: an opinion article

**DOI:** 10.1080/07853890.2024.2396075

**Published:** 2024-08-26

**Authors:** Ting Zhang, Wei Liu, Song Gao

**Affiliations:** a College of Physical Education and Health Sciences, Zhejiang Normal University, Jinhua, China; b University Hospital, Zhejiang Normal University, Jinhua, China; c Sports and Health Laboratory, College of Physical Education, Guangxi University of Science and Technology, Liuzhou, China

**Keywords:** Serum urate, hyperuricemia, gout, nutrition, exercise

## Abstract

Hyperuricaemia (HUA) is an abnormally high concentration of serum urate caused by either an excess of uric acid production or decreased excretion capacity in the body. Serum urate concentration forms sodium salts that deposit in the soft tissues of the joints, ultimately leading to gout. Additionally, HUA is strongly associated with several acute and chronic illnesses. In various clinical guidelines and practices, xanthine oxidase inhibitors, such as allopurinol and febuxostat, are commonly used as the initial medication for treating HUA. However, extended usage of urate-lowering drugs may have risks, including cardiovascular thrombotic events and hepatic impairment. Implementing a scientifically informed fitness diet in conjunction with appropriate exercise may decrease HUA. Unfortunately, there is currently a shortfall in exercise intervention trials for individuals suffering from HUA. Most of the previous evidence suggesting that exercise improves serum urate levels comes from intervention trials in other populations, and serum urate is only one of the outcomes observed. This opinion article analyses the causes of HUA, offers dietary and exercise guidance with the aim of furnishing a point of reference for individuals with HUA or fitness enthusiasts.

## Introduction

Hyperuricemia (HUA) is an excessively high blood concentration of uric acid caused by excess uric acid production or decreased excretion capacity in the body and is most common in middle-aged and elderly men and postmenopausal women [1]. Uric acid is a product of the body’s metabolism, derived from ingested purine-containing foods or from purines synthesized in the body [2]. Purines are components of nucleic acid bases and second messengers, such as the cell membrane messenger cAMP, which are mainly found in the cell nucleus, and are important components of genetic material, such as DNA [3]. Therefore, high serum urate levels are closely related to purine metabolism.

High levels of serum urate are a direct cause of gout [4]. Epidemiological statistics show that 36% of people with hyperuricemia will develop gout, and the risk is proportional to the level and duration of uric acid [5, 6]. The high concentration of serum urate concentration forms sodium salts that deposit in the soft tissues of the joints, resulting in gout, which can cause joint swelling, pain and even deformity [7]. Besides, HUA is highly correlated with several acute and chronic diseases, including metabolic syndrome, coronary heart disease, heart failure, stroke, hypertension, chronic obstructive pul disease and malignant tumors [8]. The recent evidence supports that hyperuricemia may be an independent cardiovascular risk factor [9]. Epidemiological studies have found that uric acid can independently predict hypertension, stroke, coronary heart disease and heart failure [9]. The preliminary study shows that lowering uric acid in human body can lower blood pressure of hypertensive individuals [10, 11]. In various guidelines and clinical practices, xanthine oxidase inhibitors, such as allopurinol and febuxostat, are mostly used as first-line drugs in the treatment of HUA [12, 13]. However, long-term use of urate-lowering drugs may be associated with risks such as cardiovascular thrombotic events and liver damage [14]. Non-pharmacological treatments for HUA include dietary modification, alcohol and tobacco restriction, regular exercise and weight control [15]. Studies have shown that people with HUA can reduce their serum urate concentration by 10–18% or 70–90 μmol/L through healthy lifestyle management [16].

In 2007, the American College of Sports Medicine and the American Medical Association launched the ‘Exercise is Medicine’ program [17]. The benefits of exercise for chronic diseases have recently been widely demonstrated [18]. As a non-drug therapy, exercise prescription has been widely used in the prevention and treatment of hypertension, diabetes, hyperlipidemia and other diseases [19]. Exercise prescription is to improve physical fitness, promote physical and mental health as the main goal, according to the participants’ physical strength as well as cardiovascular function status, the content, intensity, time and frequency of exercise in the form of prescription, and put forward precautions [20, 21]. Exercise prescriptions have clear immediate and long-term goals and are more planned [22]. Exercise prescriptions are developed and implemented according to the specific circumstances of each participant, and are very specific. It is important to note that people with severe heart disease, hypertension, infectious diseases, etc. should not follow an exercise prescription. If chest pain, arrhythmia, dizziness, etc. occur during exercise, the exercise should be stopped immediately [19].

Patients are eager to get fit through exercise, and even more eager to achieve a perfect body shape. More and more people are getting involved in fitness or bodybuilding, trying to participate in high-intensity strength training and over-consuming excess protein powders in their diets. However, instead of getting fit, the result is often injury, HUA or even gout [23]. Gout is a common form of inflammatory arthritis characterized by recurrent attacks of joint pain [24]. The affected joint is swollen, red and warm to touch. The first attack usually affects only one joint, most commonly the big toe, and lasts a few days [25]. Subsequent attacks may involve several joints and last for weeks with shorter intervals or remissions in between. Gout is caused by deposits of urate crystals in the joints, which can happen when serum urate levels are too high [26]. Gout doesn’t seem to get a lot of attention, yet high uric acid on a medical report is not uncommon. For most people, the primary cause of gout is the urate transporter, with overproduction accounting for a relatively minor contribution. People who take part in fitness activities have a higher risk of exercise-induced HUA. Thus, scientific education and dietary advice are therefore particularly important.

A scientifically based fitness diet combined with appropriate exercise may be effective in reducing HUA. However, there is currently a lack of exercise intervention trials in people with HUA. Most of the previous evidence that exercise improves uric acid levels comes from intervention trials in other populations, and uric acid is only one of the outcomes observed. Exercise is one of the risk factors for hyperuricemia because continuous high-intensity exercise causes lactic acid accumulation, which inhibits uric acid excretion [27, 28]. At the same time, the right training mode is also an effective means of improving hyperuricemia. For example, maintaining moderate and low intensity aerobic exercise is beneficial for reducing serum urate [29]. Additionally, exercise can reduce inflammation in the body, which can help relieve the symptoms of gout [19]. People with HUA should adjust their diet and exercise, rather than blindly eating protein powders or exercising. However, the current study does not provide a detailed exercise programme to lower uric acid. This opinion article analyzes the causes of HUA, provides guidance on diet and exercise, and recommends exercise prescriptions, aiming to provide a reference for people with HUA or fitness enthusiasts.

### Search strategy

A comprehensive literature search was conducted in PubMed, EMBASE and Web of Science using the following search terms: (hyperuricemia [Title] OR uric acid [Title] OR urate [Title] OR gout [Title]) AND (exercise [Title] OR athletics [Title] OR sport [Title] AND (randomised controlled trial [Title]). We excluded review articles and prioritised articles on RCTs with hyperuricemia as the main research method.

## HUA: serum urate metabolism imbalance

**Figure 1.**
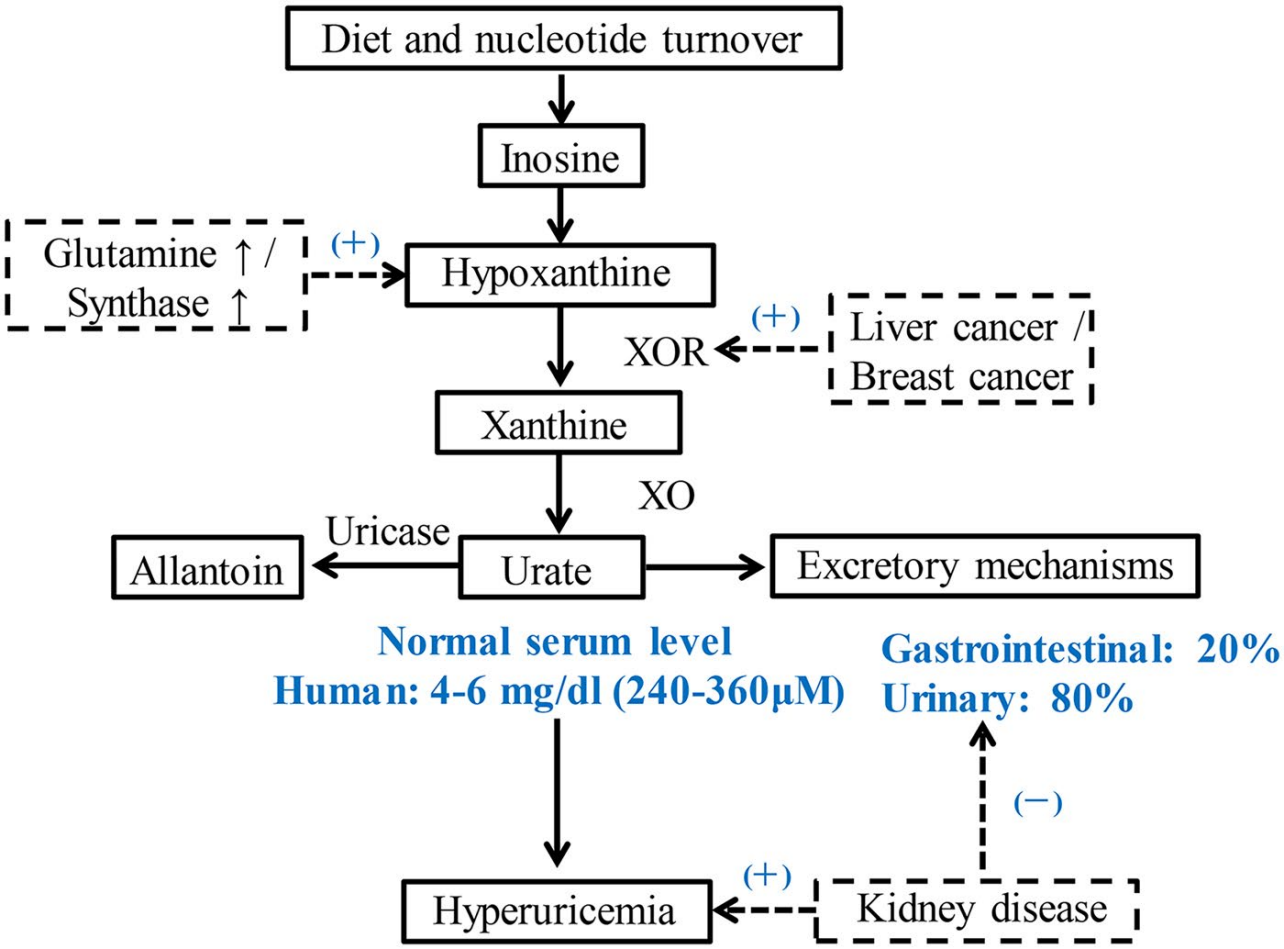
Pathways of urate homeostasis. Summary scheme of the pathways to produce uric acid, to convert it into allantoin by the liver enzyme uricase, and to excrete it. The balance between these pathways is disrupted causing hyperuricemia. XOR: xanthine oxidase reductase; XO: xanthine oxidase; (+): increase; (-): reduce.

Serum urate is the end product of purine metabolism and is formed from hypoxanthine and xanthine under the action of xanthine oxidase (XO) [30]. The average uric acid level in the normal human body is 1200 mg, with 750 mg produced and 500–1000 mg excreted daily [31]. The human body metabolizes endogenous and dietary purines to produce uric acid, which is continuously excreted by the kidneys and intestines. The production and excretion of uric acid in a healthy body can be kept within a stable range. If the body produces more uric acid than it can excrete, uric acid levels in the blood will rise. High serum urate levels can be caused by increased production or decreased excretion (Figure 1) [32].

### Increased serum urate production

Tumors, blood diseases, etc. lead to increased nucleic acid metabolism, and many purines are broken down into uric acid, resulting in HUA; The enzyme deficiency of purine reabsorption or the synthesis of purine base substrates, such as glutamine concentration is too high, resulting in excessive purine production and breakdown into uric acid. Whether it is increased nucleic acid metabolism or increased purine synthesis, it is associated with metabolic disorders [33, 34]; xanthine oxidase reductase (XOR) is a rate-limiting enzyme in the purine catabolic pathway that can oxidize hypoxanthine to xanthine, which in turn catalyzes the oxidation of xanthine to uric acid. XO shows xanthine dehydrogenase activity in the physiological state, whereas in pathological conditions it shows higher xanthine oxidase activity, ultimately leading to excessive uric acid production [35]. The above process only occurs in tissues that express xanthine oxidase, mainly including liver, small intestine and breast tissues. Studies have shown that XOR is highly expressed in breast cancer, liver cancer, gastrointestinal tumors and kidney cancer [36].

### Reduced serum urate excretion

About 20% of uric acid is broken down into ammonia and carbon dioxide in the intestine, and most of the uric acid is excreted through the kidneys. Therefore, kidney disease leads to impaired excretion and uric acid retention, resulting in HUA [37]. The uptake and secretion of uric acid by the renal tubules is influenced by specific regulatory mechanisms. Several uric acid transport proteins form a multimolecular complex that plays a key role in regulating serum urate levels, and their dysfunction will directly affect blood uric acid levels. The main protein that regulates uric acid secretion in the renal tubules is ATPbinding cassette subfamily G member 2 (ABCG2) [38]. A missense mutation in the ABCG2 gene can cause hyperuricemia and gout [39]. Notably, many drugs can interfere with renal tubular excretion of urate or increase uric acid formation, which can induce hyperuricaemia and even gout [40]. Diuretic-induced hyperuricaemia has become common in recent years. Other drugs that can cause hyperuricaemia are salicylates, pyrazinamide and ethambutol [41].

## HUA: diet and exercise

Diet and exercise are essential and effective ways of improving HUA. Normally, uric acid production and excretion should always remain in a homeostatic state. However, when high serum urate levels occur, it is necessary to strictly control the foods that can produce uric acid, starting with a low-purine diet. People with hyperuricemia should follow a low-purine diet (i.e. low-protein) diet, which consists of reducing alcohol intake, avoiding seafood, drinking more water, and eating enough fruit and vegetables [42]. Moreover, fitness enthusiasts should pay sufficient attention to HUA. Exercise can improve and treat many diseases, but long-term high-intensity exercise training or excessive high-protein diet can greatly increase the incidence of HUA [43]. Muscle energy sources such as creatine phosphate are severely depleted during vigorous exercise, which increases purine production and can increase uric acid. Additionally, uric acid crystals can be deposited in the joints of individuals with hyperuricemia [44]. Excessive exercise can lead to joint damage from urate deposition, which can trigger acute gout flares [45, 46].

### High protein intake induces HUA

Fitness enthusiasts involved in strength training for muscle building should promote muscle synthesis and prevent exercise fatigue, which requires a high-protein diet and some additional fitness supplements. This may lead to a higher purine intake and risk of elevated blood uric acid than the general population [47]. Mainly as follows:

Long-term high-protein meals such as meat, seafood and other animal protein rich diets lead to excessive purine intake and cause HUA [48]. Notably, the study found an inverse relationship between vegetable protein intake and uric acid levels [49].

Excessive supplementation with fitness nutrient, particularly glutamine, leads to an excessive concentration of substrates for de novo purine synthesis, which promotes the metabolism of many purines and causes HUA [50].

A long-term high-protein diet is essential for ‘muscle building’, but whey protein or casein powder with high biological value and low purine content should be the first choice. Lean meat, seafood or vegetable protein powders contain high-purine protein, which is 3 to 5 times higher than normal protein intake [51].

Fitness enthusiasts should pay attention to the consumption cycle of nutritional supplements, especially glutamine. Although training consumes a lot of glutamine, excess glutamine leads to increased purine synthesis.

Sports nutrition recommendations for lowering blood uric acid:

For additional high-protein supplements, choose whey protein powder; choose low-purine foods such as eggs and dairy products for daily meals, and stew lean meats and avoid soups to reduce purine intake; limit the intake of large amounts of beans, seafood and shellfish, which are high in protein and purines; use fitness nutrition products at intervals, such as 4–6 weeks after consumption, stop for 1–2 weeks, and determine the dosage strictly according to fitness training needs, especially glutamine and amino acid products. This dietary choice is based on evidence that meat, seafood, alcohol and fructose-rich foods increase the risk of hyperuricemia and gout, whereas consumption of low-purine dairy products reduces serum urate levels [52, 53].

### Fitness training induces HUA

**Figure 2.**
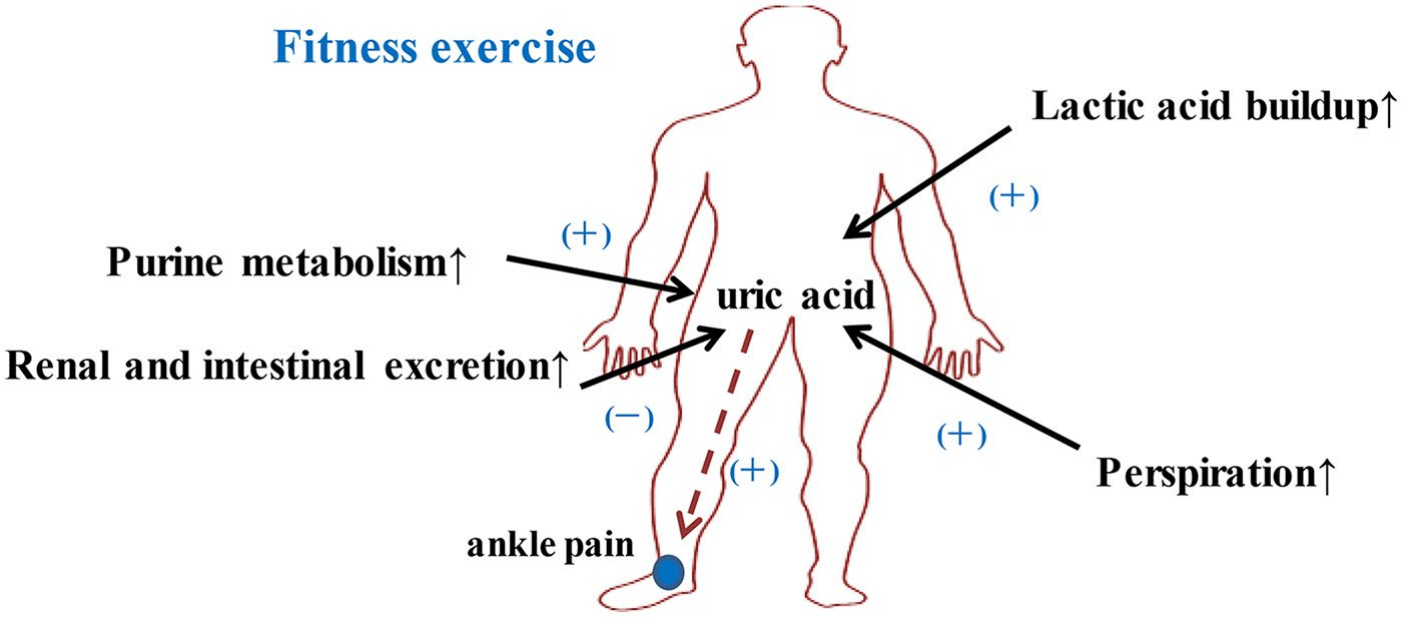
Schematic diagram of the increase in uric acid caused by fitness exercise.

Exercise can cause temporary HUA. If the serum urate is not eliminated in time and builds up over a long period, it can lead to gout. People with HUA take part in exercise to improve uric acid levels, but this may have the opposite effect. The main factors that cause HUA due to exercise are: increased metabolism, increased sweating during exercise, and lactic acid accumulation (Figure 2).

Exercise increases metabolism, including purine metabolism and renal and intestinal excretion. The increase in metabolism during exercise causes a transient increase in serum urate, but the sustained excretion of serum uric acid increases after exercise. Therefore, this is not a factor in the increase in serum urate accumulation.

Exercise causes increased sweating, while renal excretion of uric acid is significantly reduced, and a large amount of uric acid accumulates in the plasma, which is particularly pronounced during aerobic fat burning exercise. Joint pain caused by long-term aerobic fat-burning exercise is often confused with chronic joint strain, especially in the ankle. However, pain caused by uric acid accumulation does not improve after various treatments and changes in aerobic fat burning exercise, such as switching from treadmill jogging to cycling.

There are differences in the effects of different exercise intensities on serum urate concentrations. High-intensity or short-term acute exercise can lead to elevated blood uric acid, and long-term such exercise may increase the risk of hyperuricemia [54, 55]. During resistance exercise, the anaerobic mode of glycolysis leads to the accumulation of lactic acid, which inhibits uric acid excretion and causes the amount of uric acid to increase after exercise. After long-term high-intensity resistance training, the human body evolves from a transient increase in blood uric acid to a persistent hyperuric acid state [55, 56]. This is the main reason for the increase in blood uric acid caused by inappropriate exercise.

### Exercise therapy for HUA

The effectiveness of exercise in improving serum urate levels has been demonstrated in several studies [57, 58]. Yuan and his colleagues reported that a 45-day aerobic exercise program reduced participants’ serum urate levels by 10.5% (*p* < 0.05) [59]. They found that men with mild hypertension who did 8 weeks of aerobic exercise had 41.8% (*p* < 0.05) reduction in serum urate [60]. However, another study found that 12 weeks of resistance training increased blood uric acid levels in patients with type 2 diabetes (*p* < 0.001) [55]. A study by Huang et al. [23] showed that serum urate levels were significantly increased in professional athletes after short-term high-intensity exercise (*p* < 0.05). Thus, low-intensity exercise may be an appropriate option to improve serum urate levels, providing a basis for exercise prescription guidelines for HUA management.

Exercise can indeed disrupt the serum urate balance, but if the intensity, methods and precautions of exercise are mastered, exercise can still have a positive therapeutic effect on high serum urate, HUA and even gout.

Although exercise temporarily increases serum urate, the human body’s ability to metabolize serum urate increases after exercise, which generally promotes serum urate metabolism.

Exercise improves metabolism, speeds up blood circulation, slows down and reduces urate deposition in bones and joints, and hinders the conversion of high serum urate into gout.

Exercise not only improves the body’s ability to metabolize, it can also reduce weight, lower blood pressure, blood fats, and blood sugar. When treating the disease, reducing the factors that inhibit uric acid excretion, especially lowering blood lipids, will improve the kidney’s ability to metabolize uric acid in the blood.

However, exercise is a cause of increased serum urate, which has been demonstrated in high-intensity professional athletes [61]. Studies have shown that high-intensity exercise groups or athletes have a higher incidence of hyperuricemia [62, 63]. Therefore, exercise intensity, exercise elements, exercise frequency and precautions are not only important for bodybuilders and fitness enthusiasts, but also elements of exercise therapy to improve HUA.

Exercise intensity: Increased blood lactate after resistance training is one of the important factors leading to HUA, so exercise intensity is important to reduce serum urate levels. Usually, high-intensity training can select the high-phosphate training mode, while general regular training can select the mixed mode of low-intensity glycolysis metabolism + aerobic exercise. Both modes minimize lactate production during resistance training.

Aerobic fat burning training should include low intensity aerobic exercise.

Choice of exercise mode: Combining simple resistance training with aerobic fat burning can maximize the lactic acid consumption produced by exercise. Fitness enthusiasts can perform aerobic fat burning after resistance training to completely metabolize the lactic acid produced by resistance training [64]. Furthermore, this training mode helps to completely eliminate uric acid from the blood and avoid excessive accumulation caused by intense training.

Note: Special attention should be paid to timely hydration to speed up urination. This is particularly important during fat burning aerobic exercise, when the body perspires heavily and when the ambient temperature is high.

## Discussion and conclusion

The authors of this article point out that exercise can improve serum urate levels. It is an effective non-pharmacological intervention. Besides, given the discrepancy in the study results, the existing problems of exercise for HUA need to be analyzed in depth, such as the duration and intensity of exercise. This is a key issue that needs to be addressed in future studies. Furthermore, more evidence-based studies are needed to demonstrate the effectiveness of exercise in improving HUA. Researchers should fully consider whether patients with HUA have other diseases when designing exercise prescriptions.

## Data Availability

Data sharing is not applicable to this article, given that no new data were created or analysed in this study.
